# Acute Confusion as the Presenting Feature of Hypoglycemia and Lactic Acidosis in an Adult With Known Fructose-1,6-Bisphosphatase Deficiency

**DOI:** 10.7759/cureus.116754

**Published:** 2026-09-22

**Authors:** Mandek Farah Ahmed, El Mahdi Benmasbahi, Ahmed Belkouch, Lekhit Mounir, Naoufal Chouaib

**Affiliations:** 1 Emergency Medicine, Mohammed V Military Training Hospital, Rabat, MAR

**Keywords:** acute confusion, altered mental status, emergency medicine, fbpase deficiency, gluconeogenesis, hypoglycemia, inborn error of metabolism, lactic acidosis

## Abstract

We report the case of a 29-year-old woman with genetically confirmed fructose-1,6-bisphosphatase deficiency, an inherited disorder of gluconeogenesis. She had been followed since childhood by a specialized metabolic unit and was brought to the emergency department by a mobile intensive care service because of acute confusion. Her symptoms had begun the previous evening with epigastric pain, repeated vomiting, anorexia, and intense asthenia. Consequently, she had eaten almost nothing for approximately 20 hours before the onset of confusion. The Glasgow Coma Scale (GCS) score recorded during the pre-hospital assessment was 8, with a capillary blood glucose concentration of 0.4 g/L (2.2 mmol/L), which was partially corrected during transfer. On arrival, her GCS score was 10. She had no focal sensorimotor deficit, and her pupils were equal and reactive. Her respiratory rate was 26 breaths per minute, peripheral oxygen saturation was 98% on room air, blood pressure was 140/80 mmHg, and heart rate was 120 beats per minute. The abdomen was soft and non-tender. Arterial blood gas analysis showed severe metabolic acidosis with appropriate respiratory compensation (pH 7.185; bicarbonate, 6.3 mmol/L) and a lactate concentration of 6.49 mmol/L. The full blood count showed moderate leukocytosis with neutrophil and monocyte predominance without anemia. Computed tomography of the chest, abdomen, and pelvis, performed after treatment had been initiated to exclude an intercurrent precipitating condition, was normal. She was treated with intravenous 30% dextrose, followed by a continuous intravenous dextrose infusion, and was admitted to the intensive care unit. Her consciousness returned to a GCS score of 15, and the acid-base disturbance corrected in parallel. Mechanical ventilation was not required. She was discharged from the intensive care unit after 48 hours, with complete neurological recovery. This episode did not represent a new diagnosis but rather an acute decompensation of an already known metabolic disease, precipitated by vomiting and a fast of approximately 20 hours. In a patient with a known disorder of gluconeogenesis, acute confusion following vomiting or reduced oral intake should prompt consideration of a hypoglycemic and lactic acidosis crisis until proven otherwise. Bedside glucose measurement and arterial blood gas analysis can establish the diagnosis within minutes, and intravenous dextrose is the treatment for both abnormalities.

## Introduction

Fructose-1,6-bisphosphatase catalyzes the hydrolysis of fructose-1,6-bisphosphate to fructose-6-phosphate, a key irreversible step in gluconeogenesis. Baker and Winegrad first described the enzyme deficiency, reporting fasting hypoglycemia and metabolic acidosis in a child with absent hepatic enzyme activity [1]. Pagliara et al. later established the disorder's characteristic biochemical signature: hypoglycemia associated with lactic acidosis that develops when endogenous glucose production is required [2].

The functional consequence of the enzymatic block is straightforward. Glycogenolysis is not impaired, so glucose can still be released from hepatic glycogen, and short periods of fasting are usually well tolerated. Once glycogen stores are exhausted, however, glucose can no longer be synthesized from lactate, glycerol, or gluconeogenic amino acids. These precursors consequently accumulate, particularly lactate, because lactate produced continuously by peripheral tissues can no longer be taken up by the liver and recycled into glucose.

Decompensation is therefore precipitated by prolonged fasting, febrile illness, vomiting, or any reduction in oral intake and manifests as hypoglycemia accompanied by hyperlactatemia, hyperventilation, somnolence, seizures, or coma. The disorder is inherited in an autosomal recessive manner and results from biallelic variants in the FBP1 gene [3]. In a French series reported by Lebigot et al., acute metabolic crises were the presenting event in the great majority of patients, and episodes continued to occur beyond childhood in a proportion of patients [4].

Nevertheless, adults with a known diagnosis continue to present to emergency departments, where the combination of altered mental status and hyperventilation may be attributed to more common causes. In such patients, the episode is not a diagnostic problem but an acute decompensation of an already known disease, and the principal challenge is recognizing it quickly enough. We report an episode of acute confusion that was the presenting feature of a severe hypoglycemic and lactic acidosis crisis in a young woman already known to have genetically confirmed fructose-1,6-bisphosphatase deficiency. We discuss the clinical reasoning that allows this presentation to be recognized and treated without delay.

## Case presentation

A 29-year-old woman was brought to the emergency department by a mobile intensive care service because of acute confusion. She was already known to have fructose-1,6-bisphosphatase deficiency, confirmed by molecular analysis of the FBP1 gene at a specialized metabolic unit, where she had been followed since childhood. The present episode represented an acute decompensation of her known disease rather than a new diagnosis. The metabolic unit had provided her with a written, individualized emergency management plan based on an oral glucose polymer regimen while she remained well, with a switch to intravenous dextrose if oral intake could not be maintained. She was not receiving any other regular treatment and had no other medical history. The detailed molecular report was held by the referring unit and could not be retrieved for this publication; therefore, the individual FBP1 variants are not reported here.

The episode began on the evening before admission with epigastric pain, repeated vomiting, anorexia, and intense asthenia. Her oral intake had been almost nil for approximately 20 hours. Because of the repeated vomiting, she was unable to use the oral glucose polymer component of her emergency plan at home. Confusion developed the following day and worsened rapidly, prompting a call to the emergency medical service.

The Glasgow Coma Scale (GCS) score recorded during the pre-hospital assessment was 8/15, and the capillary blood glucose concentration measured at the same time was 0.4 g/L (2.2 mmol/L). Intravenous glucose was administered during transfer, with partial improvement in her level of consciousness, although the vomiting and epigastric pain persisted.

On arrival at the emergency department, the patient remained confused, with a GCS score of 10/15. There was no focal sensorimotor deficit, and the pupils were equal and reactive. She was tachypneic, with a respiratory rate of 26 breaths per minute, but was not hypoxemic; peripheral oxygen saturation was 98% while she was breathing room air. Her blood pressure was 140/80 mmHg, and her heart rate was regular at 120 beats per minute. The abdomen was soft and non-tender, with no organomegaly. She was apyrexial, with no rash, neck stiffness, or clinical focus of infection.

The peripheral oxygen saturation was concordant with the arterial blood gas sample obtained at the same time, which showed a partial pressure of oxygen of 113 mmHg and an oxygen saturation of 97% as calculated by the analyzer.

The patient did not require supplemental oxygen at any point during the admission. There was no respiratory deterioration, mechanical ventilation was never required, and computed tomography of the chest was normal. The tachypnea was therefore interpreted as respiratory compensation for the metabolic acidosis and resolved as the acidosis was corrected.

Bedside arterial blood gas analysis showed severe metabolic acidosis with significant hyperlactatemia; the results are presented in Table 1. The respiratory response was appropriate for the degree of metabolic acidosis. For a bicarbonate concentration of 6.3 mmol/L, Winter's formula predicts a partial pressure of carbon dioxide of 17.5 ± 2 mmHg (range, 15.5-19.5 mmHg), whereas the measured value was 16.6 mmHg [5]. The lactate concentration of 6.49 mmol/L accounted for a substantial part, but not the entirety, of the base deficit of 22 mmol/L. Blood ketone bodies were not measured.

**Table 1:** Arterial blood gas analysis on admission The measurement was performed at the bedside on an arterial sample at a temperature of 37.0 °C using a point-of-care analyzer (i-STAT, CG4+ cartridge). Respiratory compensation was appropriate for the degree of metabolic acidosis: Winter's formula predicts a partial pressure of carbon dioxide (PCO₂) of 1.5 × 6.3 + 8 = 17.5 ± 2 mmHg, corresponding to a range of 15.5-19.5 mmHg; the measured PCO₂ was 16.6 mmHg. The sO₂ of 97% was calculated by the analyzer from the measured PO₂ and pH, whereas peripheral oxygen saturation measured by pulse oximetry at the same time was 98% on room air. The anion gap could not be calculated because the serum electrolyte results obtained on admission were not available for this report.

Parameter (unit)	Patient value	Reference interval
pH	7.185	7.35-7.45
PCO2 (mmHg)	16.6	35-45
PO2 (mmHg)	113	80-100
BEecf (mmol/L)	-22	-2 to +2
Bicarbonate (mmol/L)	6.3	22-26
TCO2 (mmol/L)	7	23-27
sO2 (%)	97	95-100
Lactate (mmol/L)	6.49	0.5-2.0

The full blood count showed moderate leukocytosis with neutrophil and monocyte predominance, without anemia; the results are presented in Table 2.

**Table 2:** Full blood count on admission The full blood count was performed on whole blood collected in ethylenediaminetetraacetic acid using the Alinity hq analyzer. Differential leukocyte counts are reported as absolute values, with the corresponding percentages in parentheses. No microbiological investigations or inflammatory markers were available for this report; therefore, the leukocytosis is interpreted in the text as a probable stress response rather than as evidence for or against infection. MCH: mean corpuscular hemoglobin, MCHC: mean corpuscular hemoglobin concentration, MCV: mean corpuscular volume.

Laboratory test (unit)	Patient value	Reference interval
Red blood cells (×10¹²/L)	5.03	3.8-5.9
Hemoglobin (g/dL)	14.0	12-16
Hematocrit (%)	44.9	37-47
MCV (fL)	89.2	76-96
MCH (pg)	27.8	24-34
MCHC (g/dL)	31.1	31-36
Leukocytes (×10⁹/L)	12.90	3.8-11.0
Neutrophils (×10⁹/L)	9.28 (71.9%)	1.5-7.0
Lymphocytes (×10⁹/L)	1.83 (14.2%)	1.0-4.0
Monocytes (×10⁹/L)	1.70 (13.2%)	0.2-0.8
Eosinophils (×10⁹/L)	0.00 (0.0%)	<0.4
Basophils (×10⁹/L)	0.03 (0.2%)	<0.15

Management in the emergency department began with immediate correction of the hypoglycemia. Intravenous 30% dextrose was administered through a peripheral venous line, followed by a continuous intravenous dextrose infusion, together with intravenous fluid resuscitation and correction of the acid-base disturbance. Capillary blood glucose was monitored repeatedly and increased to 1.1 g/L (6.1 mmol/L). The serum potassium concentration remained within the reference interval throughout, and no local complication related to the peripheral infusion was observed. The exact volume of 30% dextrose administered and the subsequent glucose infusion rate in mg/kg/min were not documented in the medical record in a form that allowed them to be reported. The airway remained clinically protected throughout, and neither tracheal intubation nor mechanical ventilation was required.

Imaging did not delay treatment. Computed tomography of the chest, abdomen, and pelvis was requested only after the dextrose infusion had been initiated because the epigastric pain and vomiting persisted and an intercurrent thoracic or abdominal process, among the classical precipitants of decompensation in this disorder, needed to be excluded. The examination was normal. Cerebral imaging was not performed because the disturbance of consciousness was fully accounted for by the documented hypoglycemia, there were no focal neurological signs, and there was no history of head trauma. Consciousness improved with dextrose alone.

The patient was admitted to the intensive care unit for continued glucose infusion and monitoring. Her subsequent course was uneventful. Consciousness progressively improved to a GCS score of 15/15, and the compensatory hyperventilation resolved in parallel with correction of the acidosis. She was discharged from the intensive care unit after 48 hours with complete neurological recovery. The chronology of the episode, from the pre-hospital assessment to discharge from the intensive care unit, is summarized in Table 3. The values recorded at each stage of the episode are summarized in Table 4.

**Table 3:** Clinical course of the patient GCS: Glasgow Coma Scale, CT: computed tomography.

Stage		Findings
A	Pre-hospital assessment	GCS 8/15; blood glucose 0.4 g/L (2.2 mmol/L); intravenous glucose during transfer
B	Arrival in the emergency department	GCS 10/15; blood glucose 0.6 g/L (3.3 mmol/L); pH 7.185; bicarbonate 6.3 mmol/L; lactate 6.49 mmol/L; oxygen saturation 98% on room air; PO2 113 mmHg; no supplemental oxygen at any time
C	Treatment in the emergency department	Intravenous 30% dextrose through a peripheral line, then continuous infusion; blood glucose rising to 1.1 g/L (6.1 mmol/L); CT of the chest, abdomen, and pelvis, requested after treatment had been started: normal
D	Intensive care unit, first 48 hours	Compensatory hyperventilation resolving; continuous dextrose infusion; mechanical ventilation never required
E	Discharge from the intensive care unit	GCS 15/15; complete neurological recovery

**Table 4:** Values recorded at each stage of the episode Only the values shown were recorded in the patient's file. Serial measurements of pH, bicarbonate, and lactate were not repeated in a form retrievable for this report; this limitation is acknowledged in the Discussion section, and a complete biochemical timeline therefore cannot be presented. Capillary glucose was measured using a bedside glucose meter, while blood gas measurements were obtained using the point-of-care analyzer described in Table 1. GCS: Glasgow Coma Scale, NA: not available.

Stage	GCS (/15)	Blood glucose	pH	Bicarbonate (mmol/L)	Lactate (mmol/L)
Pre-hospital assessment	8	0.4 g/L (2.2 mmol/L), capillary	NA	NA	NA
Arrival in the emergency department, before the dextrose infusion	10	Partially corrected by the glucose given during transfer; 0.6 g/L (3.3 mmol/L)	7.185	6.3	6.49
After initial treatment in the emergency department	Improving	1.1 g/L (6.1 mmol/L), capillary	NA	NA	NA
Discharge from the intensive care unit, 48 hours	15	Within the reference interval, on dextrose infusion	NA	NA	NA

## Discussion

The presentation described here is the direct clinical consequence of a single enzymatic block. Because fructose-1,6-bisphosphatase catalyzes an irreversible step at the end of gluconeogenesis, its absence means that, once hepatic glycogen stores are depleted, glucose can no longer be produced from lactate, glycerol, or gluconeogenic amino acids [1]. Two simultaneous consequences explain the overall clinical picture (Figure 1). Hepatic glucose production ceases, causing the blood glucose concentration to fall. At the same time, lactate continuously produced by peripheral tissues can no longer be taken up by the liver and incorporated into gluconeogenesis. Consequently, the Cori cycle is interrupted and lactate accumulates [2]. The accumulation is not uniform across all gluconeogenic substrates, as glycerol and gluconeogenic amino acids are metabolized differently and their concentrations were not measured in our patient. However, lactate predominates and the metabolite is readily measurable at the bedside. Hypoglycemia and hyperlactatemia in this disorder are therefore not two separate problems but two manifestations of the same metabolic block, which explains why restoration of an exogenous glucose supply also corrects the acidosis.

**Figure 1:**
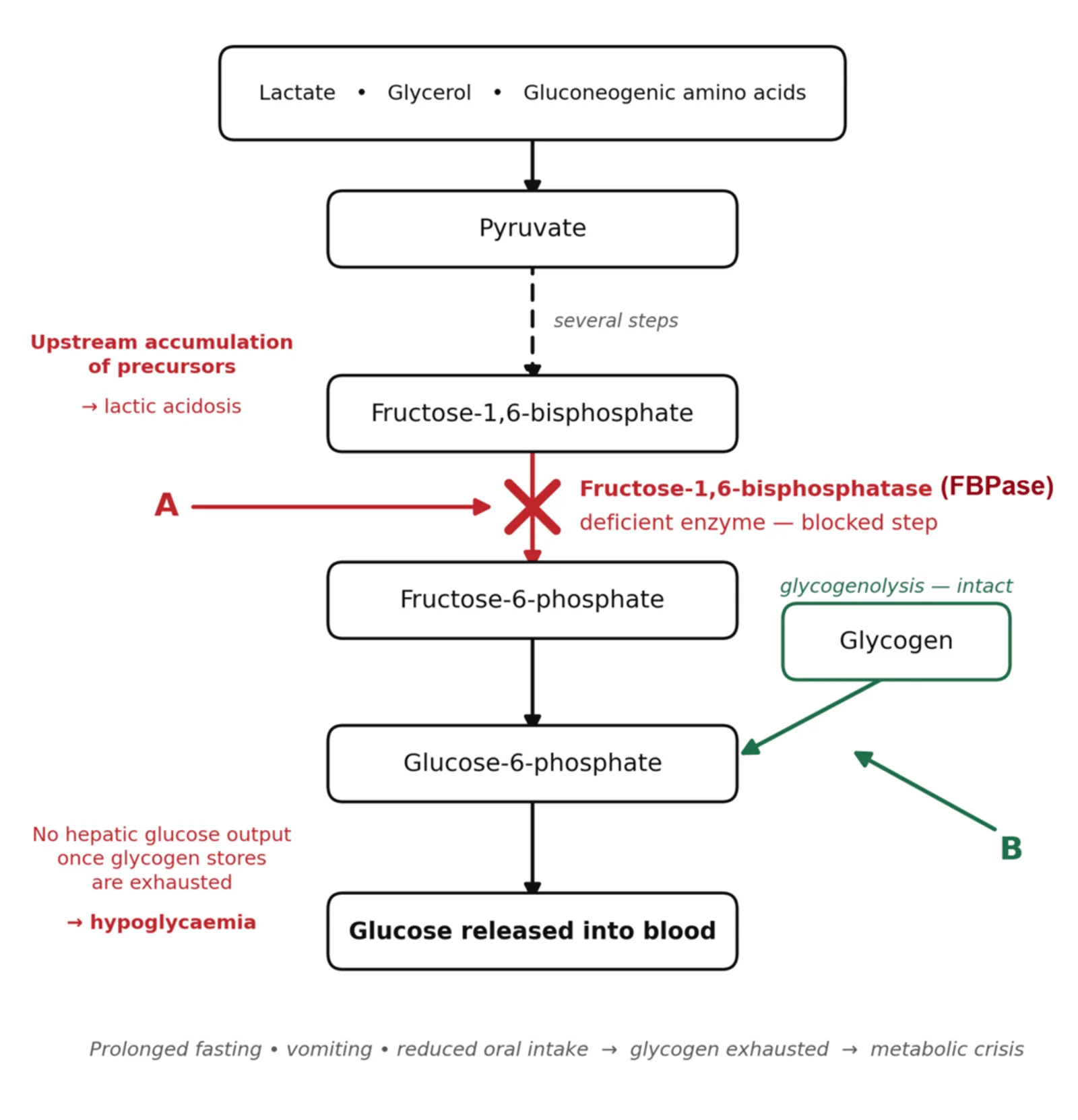
Schematic representation of the metabolic block in fructose-1,6-bisphosphatase deficiency Arrow A indicates the step catalyzed by fructose-1,6-bisphosphatase (FBPase), encoded by the FBP1 gene. In its absence, fructose-1,6-bisphosphate cannot be converted to fructose-6-phosphate, interrupting gluconeogenesis at its final step. Consequently, hepatic glucose production from gluconeogenic substrates is impaired, while lactate utilization through gluconeogenesis is also reduced, contributing to lactic acidosis. Once hepatic glycogen stores are exhausted, endogenous glucose production cannot be adequately maintained, resulting in hypoglycemia. Arrow B indicates glycogenolysis, which is not directly affected by the enzymatic defect and helps explain why short periods of fasting are usually well tolerated.

The trigger in our patient was entirely typical. An episode of vomiting accompanied by epigastric pain and anorexia had reduced her oral intake to almost nothing for approximately 20 hours, precisely the situation in which hepatic glycogen stores become depleted and gluconeogenesis becomes indispensable [3]. The clinical presentation followed the sequence described in published series: gastrointestinal prodromes, followed by asthenia, and then a rapidly progressive disturbance of consciousness [4]. It is worth emphasizing that the neurological presentation prompted the emergency call and that the gastrointestinal symptoms preceding it could easily have been attributed to uncomplicated gastroenteritis had the underlying metabolic diagnosis not been known.

The occurrence of this crisis in a 29-year-old patient with an established diagnosis deserves emphasis, as it represents the main educational point of this case. The episode was an acute decompensation of a diagnosed and followed-up metabolic disease rather than the initial recognition of the disorder, which is the clinical situation an emergency physician is more likely to encounter in an adult. Fructose-1,6-bisphosphatase deficiency is often regarded as a pediatric disorder because the first crises usually occur in infancy and tolerance to fasting generally improves with age. Nevertheless, patients who survive remain vulnerable throughout life, and decompensation can be precipitated by the same triggers, including prolonged fasting, vomiting, gastroenteritis, febrile illness, reduced oral intake, and increased metabolic demand. In the French series, metabolic crises continued to occur during adolescence and adulthood in a proportion of patients [4]. For emergency physicians, the practical implication is that fructose-1,6-bisphosphatase deficiency should remain in the differential diagnosis of an adult presenting with hypoglycemia and hyperlactatemia, in whom these findings may more commonly be attributed to sepsis, alcohol, drugs, or hepatic failure.

The acid-base findings also warrant particular attention, as they may be the feature most likely to redirect the initial diagnostic assessment. Our patient had profound metabolic acidosis, with a bicarbonate concentration of 6.3 mmol/L and a base excess of -22 mmol/L. The respiratory response was appropriate rather than maximal: the measured partial pressure of carbon dioxide of 16.6 mmHg was within the range of 15.5-19.5 mmHg predicted by Winter's formula for this bicarbonate concentration (Table 1) [5]. This compensatory response was clinically apparent as a respiratory rate of 26 breaths per minute. The lactate concentration of 6.49 mmol/L supports lactic acidosis, and in this context, the mechanism is not tissue hypoxia but impaired hepatic lactate utilization, corresponding to a type B lactic acidosis [6].

This distinction has an immediate practical consequence: correction of the underlying metabolic disturbance requires glucose rather than oxygen or vasopressor support. Oxygen and hemodynamic support remain indicated whenever genuine hypoxemia or circulatory failure is present, respectively, but they do not address the metabolic mechanism at work here. In a hemodynamically stable patient, failure of the lactate concentration to decrease should therefore prompt consideration of a metabolic rather than a circulatory cause.

The respiratory presentation also deserves specific attention because it is a potential source of diagnostic confusion. Deep compensatory Kussmaul breathing increases alveolar ventilation but does not, in itself, cause hypoxemia or oxygen desaturation. The peripheral oxygen saturation was concordant with the arterial blood gas sample obtained at the same time, which showed a partial pressure of oxygen of 113 mmHg and an oxygen saturation of 97% as calculated by the analyzer. This marked discrepancy indicates that the initial pulse-oximetry measurement was not representative of the patient's arterial oxygenation and may have been affected by peripheral factors such as vasoconstriction and marked respiratory activity. Subsequent documentation showed a peripheral oxygen saturation of 98% on room air. The hyperventilation was therefore interpreted as compensatory and resolved in parallel with correction of the acidosis. The practical corollary is that genuinely low oxygen saturation in this setting cannot be explained by the metabolic acidosis alone and should prompt investigation for an additional pulmonary cause, with aspiration or inhalation injury among the possibilities, rather than being attributed to the metabolic disturbance.

The combination of hypoglycemia and lactic acidosis has a broad differential diagnosis, and it is worth stating explicitly how alternative diagnoses were considered in this case. Sepsis was the first alternative envisaged but was judged unlikely: the patient was apyrexial and hemodynamically stable, with a normal blood pressure, no clinical focus of infection, and a normal computed tomography scan of the chest, abdomen, and pelvis. Diabetic ketoacidosis was considered unlikely in the absence of diabetes and given the presence of hypoglycemia rather than hyperglycemia. However, a degree of starvation ketosis may have contributed to the base deficit and could not be quantified because ketone bodies were not measured. A toxic or drug-related cause, particularly involving metformin, ethanol, or salicylates, was not supported by the history, and the patient was not receiving any regular medication. Acute hepatic failure and adrenal insufficiency were considered unlikely in view of the patient's complete recovery within hours following dextrose administration alone. Finally, other inherited disorders of gluconeogenesis or mitochondrial disorders can produce a similar biochemical picture; however, the pre-existing molecular diagnosis provided a sufficient explanation for the acute presentation, making further metabolic investigation unnecessary in the acute setting. Two caveats follow from this reasoning. First, a known metabolic diagnosis narrows the differential diagnosis but does not exclude a concurrent illness, which is why an intercurrent precipitating cause was actively sought after treatment had been initiated. Second, no microbiological investigations were available for this report; therefore, an infectious trigger cannot be formally excluded.

The moderate leukocytosis with neutrophil and monocyte predominance (Table 2), in an apyrexial patient with a normal computed tomography scan and no clinical focus of infection, was considered most consistent with a stress response to hypoglycemia and acidosis. 

The fact that the diagnosis had already been established at the molecular level before this admission was decisive, and it is worth recalling why molecular confirmation is not always straightforward. Santer et al. reported that a recurrent long-range deletion of FBP1 accounts for an appreciable proportion of the alleles described [7]. Subsequent studies have described correlations between genotype and residual enzyme activity, helping explain the variable severity of the phenotype [8]. A series of genetically confirmed patients has also shown that the diagnosis is frequently reached only after a prolonged evaluation aimed at excluding glycogen storage disease and mitochondrial disorders [9]. We are not in a position to report our patient's variants, their zygosity, their classification according to an accepted framework, or the technique used to detect them because the molecular report was held by the referring metabolic unit and could not be retrieved. We have therefore limited our report to stating that the diagnosis was confirmed at the molecular level by that unit and consider the absence of the detailed molecular data a limitation of this report.

Because she had a documented diagnosis and a written emergency plan, bedside glucose measurement and arterial blood gas analysis were performed within minutes of arrival, and treatment was initiated before any additional investigation (Table 3, Table 4). Emergency protocols of this kind are designed for precisely this situation. In the retrospective single-center experience reported by Rossi et al., their introduction was associated with improved prevention and management of hypoglycemia during hospital admissions [10]. Her own plan could not be implemented at home because repeated vomiting made the oral glucose polymer regimen impossible to tolerate.

Two aspects of intravenous management deserve emphasis in the emergency setting. First, glucagon is not a substitute for dextrose in this context. Although glucagon can mobilize hepatic glycogen, it does not overcome the enzymatic block in gluconeogenesis; any resulting increase in blood glucose is therefore dependent on the availability of residual glycogen stores, which may already be depleted after prolonged fasting. Second, the dextrose infusion must be continued, with appropriate electrolyte supplementation and repeated glucose monitoring, until oral intake has been reliably re-established. Premature interruption of glucose administration may expose the patient to recurrent hypoglycemia and metabolic decompensation.

Between crises, management comprises several distinct components, which are combined to varying degrees in international practice according to the patient's age, phenotype, and local practice patterns [11]. These include avoidance of prolonged fasting, with an individually defined maximum tolerated fasting interval; provision of slowly released carbohydrate, usually uncooked cornstarch, to cover the overnight period; restriction of fructose and sucrose, the degree of which varies considerably between centers; and a written emergency regimen for intercurrent illness, with oral glucose polymer while the patient is well and intravenous dextrose as soon as oral intake cannot be maintained. These components should not be presented as a single, uniform prescription.

This observation has several limitations, some of which concern data that would ordinarily have been obtained during a metabolic evaluation but were not collected during an emergency admission. Blood ketone bodies, plasma amino acids, and urinary organic acids were not measured during the crisis. The serum electrolyte results obtained on admission were not available for this report; therefore, the anion gap could not be calculated. Only the values shown in Table 3 and Table 4 were recorded, so the evolution of glucose, lactate, pH, and bicarbonate cannot be presented as a complete biochemical timeline. Serial measurements of these parameters would have been the most informative addition to the report, and Table 4 deliberately indicates which measurements are missing. The volume of 30% dextrose administered and the glucose infusion rate in mg/kg/min were not documented in a retrievable form, nor was the exact time at which oral carbohydrate and the patient's usual glucose polymer regimen were resumed. The individual FBP1 variants could not be obtained, and residual enzyme activity was not reassessed. 

## Conclusions

Acute confusion in a patient known to have a disorder of gluconeogenesis, such as fructose-1,6-bisphosphatase deficiency, should prompt immediate consideration of a hypoglycemic and lactic acidosis crisis, particularly when it follows vomiting or reduced oral intake. Capillary blood glucose measurement and arterial blood gas analysis at the bedside can identify hypoglycemia and the associated metabolic acidosis within minutes, allowing treatment to begin before neuroimaging when there are no focal neurological findings or other indications for urgent cerebral imaging. Intravenous dextrose corrects the hypoglycemia and, by restoring hepatic glucose availability, addresses the underlying metabolic disturbance responsible for the lactic acidosis. This case also demonstrates that such a crisis is not confined to childhood: it may occur in an adult whose disease has been recognized and followed since childhood, representing acute decompensation of an established disorder rather than a new diagnostic problem.

The two errors to avoid are symmetrical: attributing the disturbance of consciousness to a more common cause and delaying measurement of blood glucose, or interpreting hyperlactatemia as evidence of circulatory failure and escalating hemodynamic support without addressing the underlying metabolic defect. Oxygen and vasopressors remain indicated when hypoxemia or circulatory failure is genuinely present, and glucagon retains a role in other causes of hypoglycemia; however, neither should be considered a substitute for intravenous dextrose in fructose-1,6-bisphosphatase deficiency. When recognized promptly and treated appropriately, as in our patient, this potentially life-threatening decompensation can resolve completely.
